# Factors affecting age of onset of menopause and determination of quality of life in menopause

**DOI:** 10.4274/tjod.79836

**Published:** 2015-03-15

**Authors:** Burcu Ceylan, Nebahat Özerdoğan

**Affiliations:** 1 Ege University Faculty of Nursing, İzmir, Turkey; 2 Eskişehir Osmangazi University High School Health, Department of Midwifery, Eskişehir, Turkey

**Keywords:** Menopausal age, affecting factors, menopause specific quality of life scales

## Abstract

Menopause is a process in the climacteric period, characterized by a reduction in ovarian activity, a fall in the fertility rate, and a range of symptoms including irregular menstruation intervals. Most women enter menopause in their 40s, but this can vary from one individual to another. Although there are many factors affecting the age of menopause onset, there is no general agreement on them. Studies have shown many factors to affect the age of menopause, such as the mother’s age at menopause, the age at menarche, gestational age, use of oral contraceptives, irregular menstrual cycle, number of pregnancies, body mass index, use of tobacco and alcohol, physical activity, unilateral oophorectomy, serum lead levels, consumption of polyunsaturated fat, socioeconomic status and educational level. During this period, hormonal and biochemical changes give rise to various symptoms in the woman’s body. In menopause period, physical, psychological, social and sexual changes have a negative effect on quality of life in women. Recently, different measures have been used to assess women’s quality of life in this period of change. The purpose of this review was to examine the factors affecting the onset age of menopause and the measures of quality of life related to menopause.

## INTRODUCTION

Scientific and technological advances have lengthened the span of life and led to an increase in world’s elderly population. While in 1000 B.C., average life expectancy for women is estimated to have been 28 years of age, today this figure has reached to 8th decade of life^(1)^. According to 2013 world population data, life expectancy for newborn girls is 73 years in average around the world and 78 in Turkey^(2)^. The report of the Menopause and Osteoporosis Association of Turkey, published in 2002, reveals that the average age of menopause is 47^(3)^. Accordingly, it can be said that a woman who is expected to live 78 years spends a significant part of her life in menopause.

The word “menopause” derives from the Greek “men” (month or monthly cycle) and “pausis” (end, stop), i.e., “the cessation of monthly cycle.” The World Health Organization (WHO) describes it as the permanent cessation of menstruation as a result of the loss of ovarian follicular function^(4,5)^. The menopause signals are a reduction of ovarian activity and a fall in fertility. With the appearance of various symptoms and irregular menstrual periods, it is a characteristic phase of the climacteric stage. The hormonal and biochemical changes that occur in this period lead to various symptoms in woman’s body^(6)^.

## THE ONSET AGE OF MENOPAUSE AND INFLUENCING FACTORS

The transition from a woman’s fertile period to the period in which the ovaries begin to lose their function is achieved gradually. It is for this reason that it is difficult to set down a definite age at which menopause will begin for every woman. This period generally commences in the fourth decade of life and varies from one woman to another. Community-based studies indicate that the distribution of menopausal age displays a bell curve that ranges from age 40, ending around the age of 54, generally clustering around the ages 45-55^(7,8,9,10,11,12,13,14,15,16,17,18,19,21,22,23,24,25)^. In one study, the average age of menopause has been reported as 54 in Europe, 51.4 in North America, 48.6 in Latin America and 51.1 in Asia^(20)^.

Although there are many factors that influence the onset of menopause, there is no consensus as to whether these factors are definitive in all women (Table 1). Studies show that the onset age of menopause is affected by the age at the first menstrual period, the use of oral contraceptives, the number of pregnancies experienced, Body Mass Index (BMI), smoking, drinking alcoholic beverages, physical activity, blood lead levels and other factors^(26,27,28,29,30,31,32,33,34)^. It is believed that in about 50% of women, genetic factors play a role in determining the age of onset of menopause^(26)^. Women whose mothers entered menopause at an early age are at a high risk of early onset menopause^(27)^. In many studies, it has been shown that women who smoke enter menopause at earlier ages than non-smokers^(30,31,32,33,35,36,37,38)^. It has been observed that women who smoke 14 or more cigarettes a day enter menopause 2.8 years earlier than women who do not smoke^(39)^. Women who do not drink alcoholic beverages have been found to enter menopause at earlier ages than women who do consume alcoholic drinks^(31,33,37,38)^. While heavy physical activity is associated with early menopause^(19,33)^, light physical activity delays menopause to later ages^(31,40)^. A high consumption of polyunsaturated fats accelerates the onset of menopause while a high consumption of total calories, fruits and protein delays it(32,33). A high BMI has been found to be associated with a higher menopause onset age^(19,28,30,31,38,41,42)^. It has been found that women with hypertension and a low exposure to the sun throughout the life enter menopause at earlier ages^(19)^.

An association has been found between life-long irregular menstrual cycles and a later menopause^(30)^. An early menarche has been associated with early menopause^(27,28,29,30)^. Nulliparity has been associated with early menopause^(28,41,43)^, while multiparity is related to late menopause^(18,19,30)^. Having the first pregnancy at a later age has been associated with a later menopause onset^(32)^. The use of oral contraceptives has been associated with late menopause^(19,29,31,37)^.

Women who undergo an unilateral oophorectomy (average age=49.6 years) have been found to enter menopause at an earlier age than women who have not had the procedure (average age=50.7 years)^(42)^. A high serum ferritin level and a low bone mineral density may be the causes of early menopause^(44)^. An association has been found between onset age of menopause and bone lead levels and long-term exposure to lead (unrelated to professional reasons). According to tibia measurement results, it has been found that women with high lead levels enter menopause 1.21 years earlier than women with low levels^(34)^. Women with arsenic skin lesions have been observed to enter menopause 1.5 years earlier than women who do not have such lesions. It has been seen that women who are intensely exposed to arsenic experience menopause 2 years earlier than women who have never been exposed or who have been less exposed^(45)^.

Low socioeconomic status has been associated with early menopause^(26,28)^. Women with a lower level of education have been found to enter menopause at earlier ages than women with higher levels of education^(26,27,28,31,41,43)^.

Onset age of menopause is defined as “early menopause” when menopause commences before the age of 40^(20,46)^. Studies show that the quality of life of women who enter menopause at early ages is more adversely affected^(28,46,47)^.

## A DETERMINATION OF QUALITY OF LIFE OF WOMEN IN MENOPAUSAL PERIOD

The concept of quality of life is defined as the perception of the individual about his/her situation in life in the context of the framework of that individual’s culture and value systems, goals, expectations, standards and interests. Influenced by a complex number of factors, such as an individual’s physical health, psychological status, beliefs, social relations and environment, quality of life is used as an important measurement in assessing health status and the effects of therapies^(48)^.

In later adult years, the quality of life of women may be adversely affected by the physical and mental changes that may come about in the menopausal transition^(49)^. Quality of life in menopause is related to the degree to which a woman is able to cope with the changes and symptoms appearing in her body with the onset of menopause and her sense of satisfaction and happiness in her life during this period of transition^(50)^.

The menopausal transition is associated with physical and mental changes in a woman’s life that can have an impact on her health^(51)^. Studies show that the physical, psychological, social and sexual changes observed in the menopausal period have a negative effect on women’s quality of life^(13,14,51,52,53)^. Ninety-six percent of women have reported to experience menopause-related symptoms and their quality of life is affected not only physically and psychologically but also socially^(54,55)^. In particular, women in perimenopause and early postmenopause live through a more negative impact on their quality of life^(56)^.

Much research has been carried out on the effect of menopause on women’s quality of life and the relationship between menopause and quality of life continues to be a controversial topic^(57)^. In recent years, different instruments have been developed and used to assess women’s quality of life during the menopausal transition^(58)^. Each of these menopausal quality of life measuring instruments are of different content and they measure different aspects of life during menopause^(56)^. Although finding the ideal measuring tool for assessing quality of life is a topic still being explored, existing and accepted instruments in the literature related to menopause and their characteristics are given in Table 2a, 2b, 2c, 2d^(56,57,59,60,61,62,63,64,65)^.

Of the menopausal quality of life scales indicated in the table above, the validity and reliability of the Menopausal Rating Scale and the Menopause-Specific QOL Questionnaire have been translated into Turkish and tested in the Turkish population^(52,66,67)^. The Kupperman Index and the Greene Climacteric Scale are also used in studies in Turkey in the assessment of menopausal symptoms^(67,68,69,70,71)^. The Turkish validity and reliability studies of these two scales, however, could not be found in the literature.

## CONCLUSION

As life expectancy increases, so does the time spent in the period of menopause. Women are subjected to the hormonal and biochemical changes that adversely affect their quality of life in this period. Menopause-specific quality of life scales seek to identify and measure the severity of the menopausal symptoms women experience in order to define quality of life during menopause. The quality of life of women entering menopause at earlier ages is thought be more adversely affected. Ensuring a high quality of life for women in menopause may only be made possible by defining the extent of their quality of life and the factors related to this.

## Figures and Tables

**Table 1:**
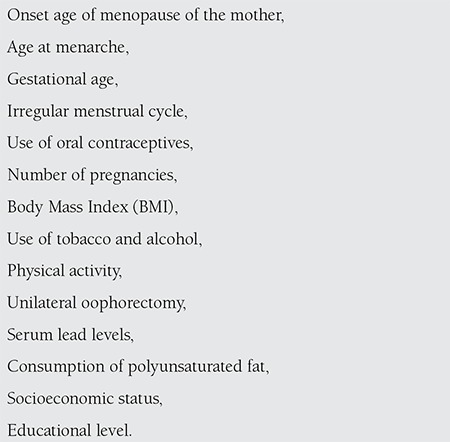
Factors affecting onset age of menopause

**Table 2a:**
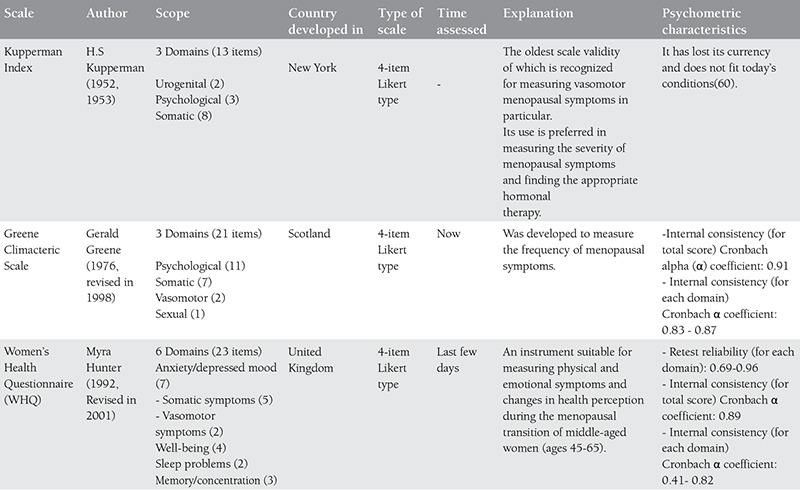
Menopausal quality of life scales and their characteristics

**Table 2b:**
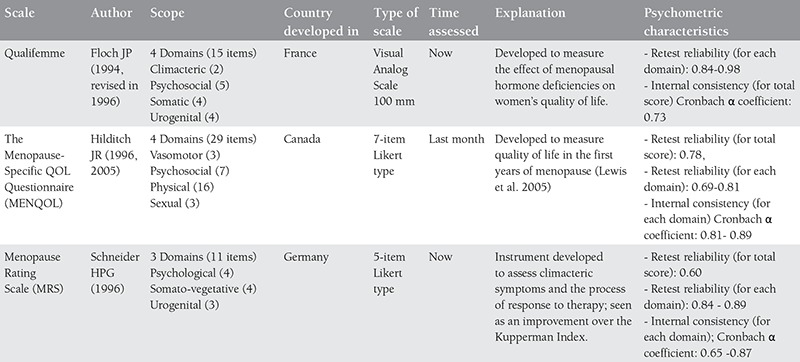
Cont’d-menopausal quality of life scales and their characteristics

**Table 2c:**
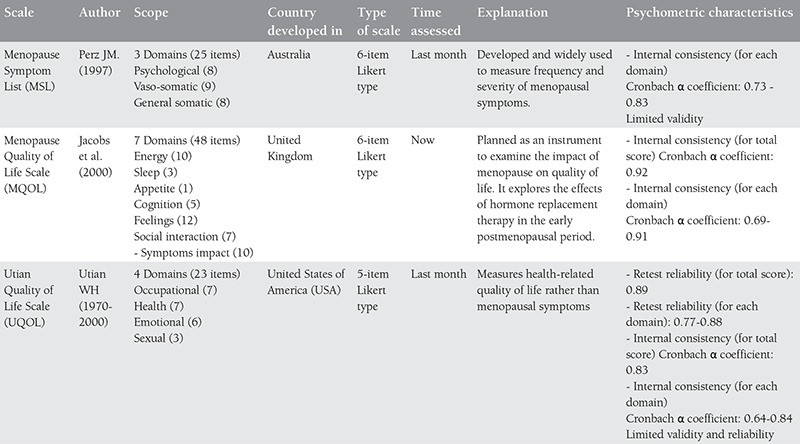
Cont’d-menopausal quality of life scales and their characteristics

**Table 2d:**
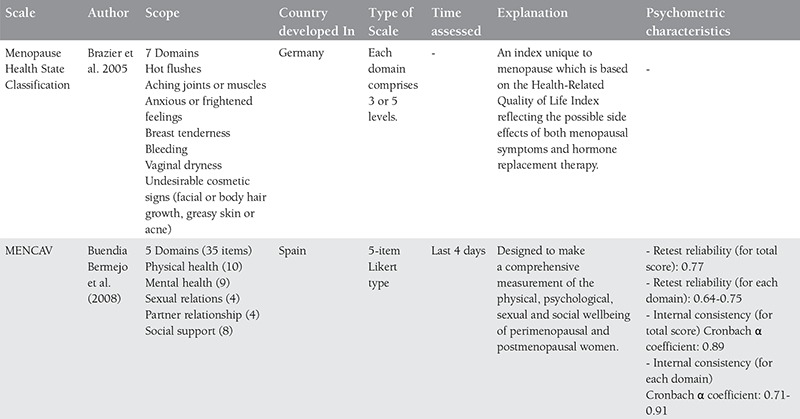
Cont’d-menopausal quality of life scales and their characteristics

## References

[1] Özkan S, Şirin A, Kavlak O (2008). Klimakteriyum ve menopoz. Kadın Sağlığı Kitabı.

[2] ((Accessed 28 Oct. 2014)). Population Reference Bureau.

[3] Ertüngealp E (2003). Türkiye Menopoz ve Osteoporoz Derneği & Türk Jinekoloji Derneği “Hormon Replasman Tedavisi” Konsensus Grubu Kararları. J Turkish German Gynecol Assoc.

[4] Şirin A (1995). Kadın ve Menopoz. Ege Üniversitesi Basımevi, İzmir.

[5] Ertüngealp E, Seyisoğlu H, Kişnişçi HA (1996). Klimakterium ve menopoz. Temel Kadın Hastalıkları ve Doğum Bilgisi.

[6] Taşkın L (2000). Doğum ve Kadın Sağlığı Hemşireliği, Sistem Ofset Matbaacılık, IV. Baskı, Ankara.

[7] Budakoğlu İ, Özcan C, Eroğlu D (2007). Quality of life and postmenopausal symptoms among women in a rural district of the capital city of the Turkey. Gynecol Endocrinol.

[8] Chen Y, Lin SQ, Wei Y, Gao HL, Wu ZL (2007). Menopause-specific quality of life satisfaction in community-dwelling menopausal women in China. Gynecol Endocrinol.

[9] Fuh J, Wang S, Lee S, Lu S, Juang K (2003). Quality of life and menopausal transition for middle-aged women on Kinmen island. Qual Life Res.

[10] Hafız I, Liu J, Eden J (2007). A quantitative analysis of the menopause experience of Indian women living in Sydney. Aust N Z J Obstet Gynaecol.

[11] Liu J, Eden JA (May). The menopausal experience of Greek women living in Sydney. Menopause 2008.

[12] Mishra GD, Brown WJ, Dobson AJ (2003). Physical and mental health: Changes during menopause transition. Qual Life Res.

[13] Peeyananjarassri K, Cheewadhanaraks S, Hubbard M, Zoa Manga R, Manocha R, Eden J (2006). Menopausal symptoms in a hospital-based sample of women in southern Thailand. Climacteric.

[14] Syed Alwi SA, Lee PY, Awi I, Malik PS, Haizal MN (2009). The menopausal experience among indigenous women of Sarawak, Malaysia. Climacteric.

[15] Tokuç B, Kaplan PB, Balık GÖ, Gül H (2006). Trakya üniversitesi hastanesi menopoz polikliniğine başvuran kadınlarda yaşam kalitesi. Trakya Üniversitesi Tıp Dergisi.

[16] Waidyasekera H, Wijewardena K, Lindmark G, Naessen T (2009). Menopausal symptoms and quality of life during the menopausal transition in Sri Lankan women. Menopause.

[17] Williams RE, Levine KB, Kalilani L, Lewis J, Clark RW (2009). Menopause-specific questionnaire assessment in US population-based study shows negative impact on Health-related quality of life. Maturitas.

[18] Abdollahi AA, Qorbani M, Asayesh H, Rezapour A, Noroozi M, Mansourian M, et al (2013). The menopausal age and associated factors in Gorgan, Iran. Med J Islam Repub Iran.

[19] Aydın ZD, Erbaş B, Karakuş N, Aydın O, Özkan ŞK (2005). Sun exposure and age at natural menopause: A cross-sectional study in Turkish women. Maturitas.

[20] Palacios S, Henderson VW, Siseles N, Tan D, Villaseca P (2010). Age of menopause and impact of climacteric symptoms by geographical region. Climacteric.

[21] Çoban A, Nehir S, Demirci H, Özbaşaran F, İnceboz Ü (2008). Klimakterik dönemdeki evli kadınların eş uyumları ve menopoza ilişkin tutumlarının menopozal yakınmalar üzerine etkisi. Fırat Üniversitesi Sağlık Bilimleri Dergisi.

[22] Çaylan A, Aydemir I, Dağdeviren N, Aktürk Z, Set T, Öztora S, et al (2011). Evaluation of health related quality of life among perimenopausal Turkish women. Health Med.

[23] Yanıkkerem E, Koltan SO, Tamay AG, Dikayak Ş (2012). Relationship between women’s attitude towards menopause and quality of life. Climacteric.

[24] Yangın HB, Kukulu K, Sözer GA (2010). The perception of menopause among Turkish women. J Women Aging.

[25] ((Accessed 08 Aug. 2014)). Türkiye Nüfus ve Sağlık Araştırması 2008.

[26] Canavez FS, Werneck GL, Parente RC, Celeste RK, Faerstein E (2011). The association between educational level and age at the menopause: a systematic review. Arch Gynecol Obstet.

[27] Özdemir O, Çöl M (2004). The age at menopause and associated factors at the health center area in Ankara, Turkey. Maturitas.

[28] Li L, Wu J, Pu D, Zhao Y, Wan C, Sun L, et al (2012). Factors associated with the age of natural menopause and menopausal symptoms in Chinese women. Maturitas.

[29] Reynolds RF, Obermeyer CM (2003). Correlates of the age at natural menopause in Morocco. Ann Hum Biol.

[30] Parazzini F, Progetto Menopausa Italia Study Group (2007). Determinants of age at menopause in women attending menopause clinics in Italy. Maturitas.

[31] Gold EB, Crawford SL, Avis NE, Crandall CJ, Matthews KA, Waetjen LE, et al (2013). Factors related to age at natural menopause: longitudinal analyses from SWAN. Am J Epidemiol.

[32] Nagel G, Altenburg HP, Nieters A, Boffetta P, Linseisen J (2005). Reproductive and dietary determinants of the age at menopause in EPIC-Heidelberg. Maturitas.

[33] Sapre S, Thakur R (2014). Lifestyle and dietary factors determine age at natural menopause. J Midlife Health.

[34] Eum KD, Weisskopf MG, Nie LH, Hu H, Korrick SA (2014). Cumulative lead exposure and age at menopause in the Nurses’ Health Study cohort. Environ Health Perspect.

[35] Butts SF, Sammel MD, Greer C, Rebbeck TR, Boorman DW, Freeman EW (2014). Cigarettes, genetic background and menopausal timing: the presence of single nucleotide polymorphisms in cytochrome P450 genes is associated with increased risk of natural menopause in European-American smokers. Menopause.

[36] Hayatbakhsh MR, Clavarino A, Williams GM, Sina M, Najman JM (2012). Cigarette smoking and age of menopause: A large prospective study. Maturitas.

[37] Stepaniak U, Szafraniec K, Kubinova R, Malyutina S, Peasey A, Pikhart H, et al (2013). Age at natural menopause in three central and eastern European urban populations: the HAPIEE study. Maturitas.

[38] Morris DH, Jones ME, Schoemaker MJ, McFadden E, Ashworth A, Swerdlow AJ (2012). Body mass index, exercise, and other lifestyle factors in relation to age at natural menopause: analyses from the breakthrough generations study. Am J Epidemiol.

[39] Kinney A, Kline J, Levin B (2006). Alcohol, caffeine and smoking in relation to age at menopause. Maturitas.

[40] Gudmundsdottir SL, Flanders WD, Augestad LB (2013). Physical activity and age at menopause: the Nord-Trøndelag population-based health study. Climacteric.

[41] Pérez-Alcalá I, Sievert LL, Obermeyer CM, Reher DS (2013). Cross cultural analysis of factors associated with age at natural menopause among Latin-American immigrants to Madrid and their Spanish neighbors. Am J Hum Biol.

[42] Bjelland EK, Wilkosz P, Tanbo TG, Eskild A (2014). Is unilateral oophorectomy associated with age at menopause? A population study (the HUNT2 Survey). Hum Reprod.

[43] OlaOlorun F, Lawoyin T (2009). Age at menopause and factors associated with attainment of menopause in an urban community in Ibadan, Nigeria. Climacteric.

[44] Lee BK, Kim Y (2014). Menopause may be the common link that resulted in the association between higher serum ferritin level and lower bone mineral density in women ≥ 45 years of age. Osteoporos Int.

[45] Yunus FM, Rahman MJ, Alam MZ, Hore SK, Rahman M (2014). Relationship between arsenic skin lesions and the age of natural menopause. BMC Public Health.

[46] Shuster LT, Rhodes DJ, Gostout BS, Grossardt BR, Rocca WA (2010). Premature menopause or early menopause: long-term health consequences. Maturitas.

[47] Benetti-Pinto CL, Almeida DM, Makuch MY (2011). Quality of life in women with premature ovarian failure. Gynecol Endocrinol.

[48] Başaran S, Güzel R, Sarpel T (2005). Yaşam kalitesi ve sağlık sonuçlarını değerlendirme ölçütleri. Romatizma.

[49] Elavsky S (2009). Physical activity, menopause, and quality of life: the role of affect and self-worth across time. Menopause.

[50] Kharbouch SB, Şahin NH (2005). menopozal dönemlerdeki yaşam kalitesinin belirlenmesi, İstanbul Üniversitesi Sağlık Bilimleri Enstitüsü Doğum ve Kadın Hastalıkları Hemşireliği Anabilim Dalı.

[51] Fallahzadeh H (2010). Quality of life after the menopause in Iran: a population study. Qual Life Res.

[52] Kharbouch SB, Şahin NH (2007). Menopozal dönemlerdeki yaşam kalitesinin belirlenmesi. İ.Ü.F.N. Hem. Derg.

[53] Som N, Ray S (2012). Menopause-specific quality of life of urban women in West Bengal, India. Menopause International.

[54] Azevedo Guimarães AC, Baptista F (2011). Influence of habitual physical activitym on the symptoms of climacterium/menopause and the quality of life of middle-aged women. International Journal of Women’s Health.

[55] İnceboz Ü, Demirci H, Özbaşaran F, Çoban A, Nehir S (2010). Factors affecting the quality of life in climacteric women in Manisa region. Trakya Üniv Tıp Fak Derg.

[56] Shin H, Shin HS (2012). Measurement of quality of life in menopausal women: A systematic review. West J Nurs Res.

[57] Zöllner YF, Acquadro C, Schaefer M (2005). Literature review of instruments to assess health-related quality of life during and after menopause. Qual Life Res.

[58] Shin H (2012). Comparison of quality of life measures in Korean menopausal women. Research in Nursing & Health.

[59] Mishra GD, Kuh D (2010). Quality of life measures during the menopause, In: Preedy VR, Watson RR, eds. Handbook of Disease Burdens and Quality of Life Measures.

[60] Schneider HPG, MacLennan AH, Feeny D (2008). Assessment of health-related quality of life in menopause and aging. Climacteric.

[61] Girod I, Loge C, Keininger D, Hunter MS (2006). Development of a revised version of the Women’s Health Questionnaire. Climacteric.

[62] Buendıa Bermejo J, Valverde Martinez JA, Romero Saiz A, Ulla Diez SM, Cobo Rodrigo A, Martinez Vizcaino V (2008). Validation of a menopause quality of life scale: The MENCAV scale. Maturitas.

[63] Brazier JE, Roberts J, Platts M, Zoellner YF (2005). Estimating a preference-based index for a menopause specific health quality of life questionnaire. Health Qual Life Outcomes.

[64] Chen RQ, Davis SR, Wong CM, Lam TH (2010). Validity and cultural equivalence of the standard Greene Climacteric Scale in Hong Kong. Menopause.

[65] ((Accessed 10 July 2014)). Dennerstein L, Guthrie J, Birkhäuser M, Sherman S. Symptoms and The Menopause.

[66] Gürkan ÖC (2005). Menopoz semptomları değerlendirme ölçeğinin Türkçe formunun güvenirlik ve geçerliliği. Hemşirelik Forumu Dergisi.

[67] Bekiroğlu N, Gürbüz A, Konyalıoğlu R, Ayas S, Alkan A, Eren S (2008). Menopoz Semptomlarını Değerlendirme Ölçeği (MSDÖ), Kupperman Menopoz Ölçeği (KMÖ) ve Nothingam Sağlık Profili (NSP) ölçeklerinin güvenilirlik ve yanıtlama etki büyüklüklerinin yeni menopozlu hastalarda karşılaştırılması. Zeynep Kamil Tıp Bülteni.

[68] Varma GS, Karadağ F, Oğuzhanoğlu NK, Özdel O, Kökten S (2006). Menopoz: Klimakterik belirtiler ve cinsel doyum arasındaki ilişki. New/Yeni Symposium Journal.

[69] Api M, Ünal O, Ağaoğlu C (1998). Hormon Replasman Tedavisinin Kupperman İndeksi, Serum Östradiol Düzeyi ve Hipoöstrojenemik İndeks İle Monitorizasyonu. Türkiye Klinikleri J Gynecol Obst.

[70] Karşıdağ AYK, Çamlıyer NA, Büyükbayrak EE, Kars B, Pirimoğlu M, Ünal O, et al (2010). Osteopenik Postmenopozal Kadınlarda Düşük Dozlu Hormon Tedavisi ve Raloksifen’in Lipid Profili, Glikoz Metabolizması ve Tiroid Hormonları Üzerine Etkilerinin Karşılaştırılması. Kartal Eğitim ve Araştırma Hastanesi Tıp Dergisi.

[71] Öztürk CŞ, Güneş H (2007). Menopozla İlişkilendirilen Belirtiler Üzerine Psikodramanın Etkisi. Jinekoloji ve Obstetrik Dergisi.

